# End Stage Renal Disease Predicts Increased Risk of Death in First Degree Relatives in the Norwegian Population

**DOI:** 10.1371/journal.pone.0165026

**Published:** 2016-11-09

**Authors:** Rannveig Skrunes, Einar Svarstad, Anna Varberg Reisæter, Hans-Peter Marti, Bjørn Egil Vikse

**Affiliations:** 1 Department of Medicine, Haukeland University Hospital, Bergen, Norway; 2 Department of Clinical Medicine, University of Bergen, Bergen, Norway; 3 Department of Transplantation Medicine, Rikshospitalet, Oslo University Hospital, Oslo, Norway; 4 Department of Medicine, Haugesund Hospital, Haugesund, Norway; The University of Tokyo, JAPAN

## Abstract

**Background:**

Increased risk of end stage renal disease (ESRD) and death in Norwegian living kidney donors has been reported, most of the donors were related to the recipient. The present study investigates risk of death in first degree relatives of ESRD patients.

**Methods:**

The Norwegian Population Registry, The Norwegian Cause of Death Registry and the Norwegian Renal Registry were linked. All citizens born in Norway, alive in 1960 and with at least one registered first degree relative were included; individuals who died during the first year of life were excluded. A cohort-design was used, ESRD in a first degree relative was the main exposure variable and death and causes of death were the main outcome variables. Cox regression statistics were used to investigate mortality risks.

**Results:**

5 130 600 individuals were included, 27 508 had at least one first degree relative with ESRD. 828 022 died during follow-up, of whom 4105 had a first degree relative with ESRD. Adjusted hazard ratio (aHR) for death was 1.13 (1.09–1.16) in individuals with a relative with ESRD compared to those without a relative with ESRD. Excluding known hereditary renal disease, aHR decreased to 1.12 (1.09–1.15). Cardiovascular death aHR was 1.15 (1.10–1.21), of which cerebrovascular death 1.34 (1.22–1.50). aHR for death due to non-hereditary renal/ureteric disease was 2.29 (1.81–2.91) with renal failure 1.80 (1.26–2.56) and glomerular disease 5.69 (3.88–8.34) as main contributors. Diabetes mellitus death aHR was 1.68 (1.35–2.10). Absolute mortality risks increased most for the oldest cohorts with excess mortality of 148 per 100.000 person years for the cohort born 1920–39 and 218 for the cohort born 1900–1919.

**Conclusions:**

ESRD in first degree relatives was associated with increased hazard ratio for death. Death due to cardiovascular disease, renal disease and diabetes mellitus increased the most.

## Introduction

A registry based study from Utah has previously described increased risk of dying with end stage renal disease (ESRD) or chronic kidney disease (CKD) in first degree relatives of ESRD patients. Relative risk of death with ESRD was 10.1 and relative risk of death with CKD was 3.89[1]. We have previously shown that first degree relatives of ESRD patients are at increased risk of developing ESRD, arguing for hereditary contributions to ESRD risk[2]. Chronic kidney disease and ESRD are well known risk factors for cardiovascular disease[3, 4], and absolute risk of death has been found to increase exponentially with declining kidney function[5]. A case control study showed that relative risk of cardiovascular death in first degree relatives of ESRD patients was 1.10 [6], with acute myocardial infarction and heart failure as the main contributors.

Increased risk of all cause death and cardiovascular death has been reported in a cohort of 1900 Norwegian living kidney donors, most of the donors were first degree relatives of the recipient[7]. A loss of three years of life compared to matched healthy controls was found in the same cohort[7]. The cause of this observation is uncertain, but may be related to reduced kidney function after nephrectomy, or genetic or environmental factors shared with the kidney recipient. Few studies have however investigated cause-specific mortality at a population-level in relatives of patients with ESRD, and more data are needed.

In the present study we link data from the Norwegian Population Registry, Norwegian Nephrology Registry and Norwegian Cause of Death Registry, and analyze whether first degree relatives of patients with ESRD have increased mortality. Risk of death was further analyzed according to cause of death as well as cause of ESRD in the relative.

## Materials and Methods

Approval from the Regional Ethics Committee was obtained before the Norwegian Population Registry, the Norwegian Cause of Death Registry and the Norwegian Renal Registry were linked to generate a de-identified dataset.

The Norwegian Cause of Death Registry was established in 1951, registering data on causes of death using the International Classification of Diseases (ICD). Primary and contributing causes of death are reported on a death certificate by the responsible physician. ICD versions 8–10 were used, version 8 for 1969–1985, version 9 for 1986–1995 and version 10 for 1996–2008. The attending physician gives the mandatory primary cause of death on the death certificate. Cause of death is later re-evaluated at the National Cause of Death Registry, using international standards. In addition to the primary cause of death, up to 3 contributing causes could have been recorded from 1969 to 1995, and up to 6 contributing causes from 1996 onwards. Data were available from January 1969 through 2008. The Norwegian Population Registry was established in 1960, identifying all Norwegian citizens and non-national residents through an 11-digit personal identification number. Registration of parental information for individuals residing with their parents started in 1970, and for this study parental information was available for most individuals born 1953 or later. The Norwegian Renal Registry has since 1980 registered all individuals in Norway with end-stage renal disease (ESRD), defined as either chronic dialysis treatment or renal transplantation. The cause of ESRD was reported by the treating physician, using the old European Renal Association-European Dialysis and Transplant Association classification[8]. Data were available through June 2009.

First degree relatives, defined as parents, siblings (defined as having the same mother and father), or children, were identified based on data from the Norwegian Population Registry. Individuals who were born in Norway and were alive in 1960 were eligible for inclusion. Individuals who died during the first year of life or with no reported first degree relatives were excluded. Data from the registries were linked using the national 11-digit identification number.

### Explanatory Variables

Development of ESRD in a first degree relative before July 2009 was the main exposure variable. A person with ESRD could be registered as a first degree relative of several included individuals.

In adjusted analyses, gender, birth year and number of recorded first degree relatives were used. Numbers of relatives were grouped as 1–2, 3–4, 5–7 and 8 or more and were included in the adjusted analysis as a continuous variable.

### Outcome Variables

**Fig 1:**
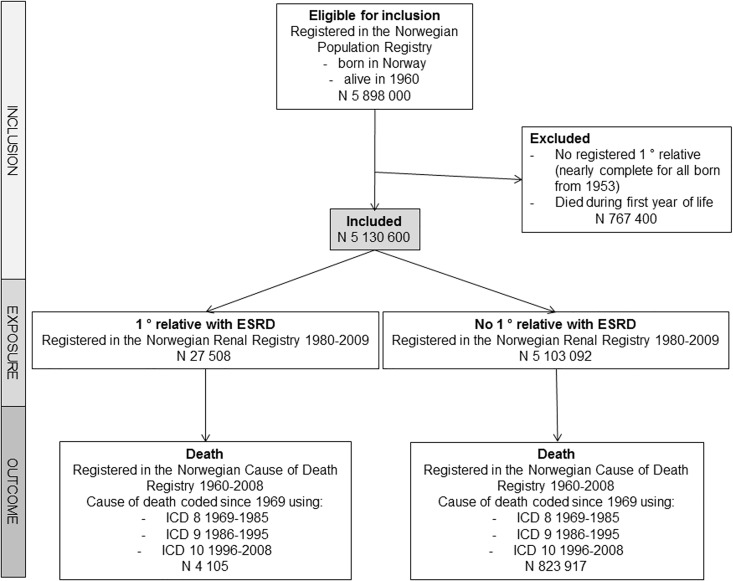
Overview of inclusion and exclusion criteria, exposure and outcomes.

The main outcome was death. Surviving individuals were followed until June 2009. Secondary outcomes were different primary causes of death. S1 Table describes the categories of cause of death that have been used in the present publication, and their ICD codes. Fig 1 describes inclusion, exposure and outcomes in a flowchart.

### Statistical Analyses

We applied a cohort design with ESRD in a first degree relative as the main exposure variable and death, or cause of death, as the outcome variables. Relative risk of death was analyzed by Cox regression analyses with age as the time variable. Analyses were adjusted by including the described variables as covariates in the Cox regression model. As cause of death was not available before 1969, the statistical analyses of cause of death were left truncated. Consequently the counting process of proportional hazards (Cox regression) was applied[9], whereby individuals are not included in the analysis until an event can be registered. Failure curves are presented as figures. Multivariate Cox proportional-hazards modeling was used to adjust for birth year, number of relatives and gender. Overall and cause-specific mortality rates were calculated as number of deaths per 100.000 person-years at risk; only follow-up years after 1969 were included in these calculations. These results are presented separately for birth year cohorts and increase in absolute mortality risk between study groups were calculated as the absolute difference between groups.

If not otherwise stated, means ± standard deviations or estimates (95% confidence intervals [95% CIs]) are given. The analyses were performed with the statistical package STATA MP edition 11.1 (StataCorp).

### Results

The study included 5 130 600 individuals, of whom 27 508 had at least one first degree relative with ESRD. During follow-up 828 022 individuals died, 4105 of these had a first degree relative with ESRD. The most common causes of death were cardiovascular disease, cancer and pulmonary disease (S1 Table). Deceased individuals in the cohort were more often men, and had fewer recorded first degree relatives than individuals who did not die during follow-up (Table 1).

**Table 1:** Characteristics of the population.

Characteristics	Total cohort		Born 1953 or later	
	Alive	Deceased	Alive	Deceased
N (%)	4 302 578 (83.9%)	828 022 (16.1%)	3 165 259 (98.4%)	51 897 (1.6%)
N (%) with first degree relative with ESRD	23 403 (0.5%)	4105 (0.5%) ^*^	17 645 (0.6%)	507 (1.0%) ^*^
Gender (% male)	2 153 680 (50.1%)	484 048 (58.5%) ^*^	1 615 444 (51.0%)	35 331 (68.1%) ^*^
Mean (SD) birth year	1969 (±23.1)	1923 (±15.8) ^*^	1980 (±16.0)	1965 (±10.4) ^*^
Mean (SD) no. of recorded relatives	4.3 (2.0)	2.5 (1.6) ^*^	4.6 (2.0)	4.5 (1.9) ^a^
Number of recorded relatives				
N (%) with 1–2 recorded relatives	721 164 (16.8%)	515 861 (62.3%) ^*^	305 033 (9.6%)	5 199 (10.0%) ^*^
N (%) with 3–4 recorded relatives	1 900 922 (44.2%)	225 287 (27.2%) ^*^	1 487 660 (47.0%)	23 807 (45.9%) ^a^
N (%) with 5–7 recorded relatives	1 366 179 (31.8%)	74 205 (9.0%) ^*^	1 120 839 (35.4%)	19 259 (37.1%) ^*^
N (%) with 8 or more recorded relatives	314 313 (7.3%)	12 669 (1.5%) ^*^	251 727 (8.0%)	3 596 (6.9%) ^*^
N (%) with recorded parents	3 491 244 (81.1%)	106 416 (12.9%) ^*^	3 119 781 (98.6%)	50 245 (96.8%) ^*^
N (%) with recorded siblings	2 999 423 (69.7%)	80 473 (9.7) ^*^	2 721 346 (86.0%)	44 279 (85.3%) ^*^
N (%) with recorded children	2 445 381 (56.8%)	745 112 (90.0) ^*^	1 390 979 (44.0%)	18 125 (34.9%) ^*^
Year of birth				
N (%) born 1900–1929	216 099 (5.0)	616 927 (74.5)	NA	NA
N (%) born 1930–1949	756 427 (17.6)	148 408 (17.9)	NA	NA
N (%) born 1950–1969	1 195 273 (27.8)	47 496 (5.7)	1 030 480 (32.6)	36 706 (70.7)
N (%) born 1970–2009	2 134 779 (49.6)	15 191 (1.8)	2 134 779 (67.4)	15 191 (29.3)

* = p <0.001

^a^ = not significant

Having a first degree relative with ESRD was significantly associated with increased risk of death. Hazard ratio (HR) for death was 1.07 (1.04–1.11) as compared to individuals without a first degree relative with ESRD (Table 2). When adjusted for gender, birth year and number of first degree relatives, HR increased to 1.13 (1.09–1.16). HR was smaller, but still highly statistically significant, after excluding individuals who had relatives with known hereditary renal disease; in these analyses, HR was 1.06 (1.02–1.09) in unadjusted analyses and 1.12 (1.08–1.16) in adjusted analyses. When individuals who developed ESRD and individuals with a relative with known hereditary renal disease were excluded, HR was 1.05 (1.02–1.09) and adjusted HR (aHR) 1.11 (1.08–1.15).

**Table 2:** Risk of death due to selected causes according to whether or not a first degree relative had ESRD.

	Relative with ESRD	N total cohort	N deceased Individuals	Hazard ratio	p-value	Adjusted hazard ratio*	p-value
Death, all causes	No	5 103 092	828 022	1.0 (ref)		1.0 (ref)	
	Yes	27 508	4105	1.07 (1.04–1.11)	<0.001	1.13 (1.09–1.16)	<0.001
Death due to cardiovascular disease	No	5 103 092	338 310	1.0 (ref)		1.0 (ref)	
	Yes	27 508	1 651	1.11 (1.06–1.16)	<0.001	1.15 (1.10–1.21)	<0.001
Death due to diseases of the kidneys and ureters	No	5 103 092	7 095	1.0 (ref)		1.0 (ref)	
	Yes	27 508	69	2.18 (1.72–2.77)	<0.001	2.35 (1.85–2.99)	<0.001
Death due to diabetes mellitus	No	5 103 092	10 973	1.0 (ref)		1.0 (ref)	
	Yes	27 508	81	1.59 (1.28–1.98)	<0.001	1.68 (1.35–2.10)	<0.001
Death due to chronic lower respiratory tract diseases and respiratory tract infections	No	5 103 092	27 988	1.0 (ref)		1.0 (ref)	
	Yes	27 508	114	0.94 (0.79–1.14)	0.54	1.00 (0.83–1.20)	0.96
Death due to cancer	No	5 103 092	223 122	1.0 (ref)		1.0 (ref)	
	Yes	27 508	951	0.93 (0.87–0.99)	0.03	0.98 (0.91–1.04)	0.45

*Adjusted for sex, birth year and no. of relatives. Individuals with diabetes mellitus as a contributing cause of death were excluded in adjusted analyses.

**Fig 2:**
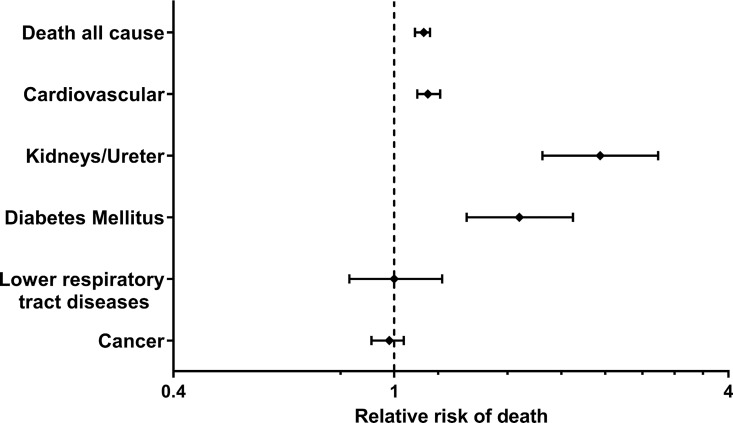
Adjusted risk of death according to different causes of death in individuals with first degree relatives with ESRD.

Hazard ratios for the most important causes of death were analyzed (Table 2), the selection was based on the three most common causes of death and causes of death known to be related to kidney disease. In these analyses, HR was significantly increased for cardiovascular death (1.11 (1.06–1.16)), death due to diseases of the kidney and ureter (HR 2.18 (1.72–2.77)) and death due to diabetes mellitus (HR 1.59 (1.28–1.98)). HR for death due to cancer was < 1 in unadjusted analyzes, and not significantly raised in adjusted analyses. These results are summarized in a Forest plot (Fig 2).

Hazard ratios for subgroups of cardiovascular death were further analyzed in Table 3, cerebrovascular death was associated with the highest adjusted HR 1.34 (1.22–1.47), aHR for death due to ischemic heart disease was 1.11 (1.03–1.18), aHR for death due to other non-valvular cardiovascular disease was 1.16 (1.02–1.34) and aHR for death due to hypertension was 1.30 (0.97–1.76). Hazard ratios for death due to subgroups of diseases of the kidneys and ureters are listed in Table 4. Diseases of the kidneys and ureters, hereditary or non-hereditary, were listed as the primary cause of death in 123 individuals with a first degree relative with ESRD, aHR was 3.95 (3.30–4.72). After exclusion of known congenital or hereditary causes, aHR decreased to 2.29 (1.81–2.91). aHR for death due to acute or chronic renal failure was 1.80 (1.26–2.56), and aHR for death due to glomerular disease was 5.69 (3.88–8.34).

**Table 3:** Risk of death due to categories of cardiovascular disease, according to whether or not a first degree relative had ESRD.

	Relative with ESRD	N total cohort	N deceased individuals	Hazard ratio	p-value	Adjusted hazard ratio*	p-value
Death due to cerebrovascular disease	No	5 103 092	77 000	1.0 (ref)			
	Yes	27 508	435	1.29 (1.17–1.41)	<0.001	1.34 (1.22–1.47)	<0.001
Death due to ischemic heart disease	No	5 103 092	180 801	1.0 (ref)			
	Yes	27 508	828	1.04 (0.97–1.11)	0.26	1.11 (1.03–1.18)	0.004
Death due to other cardiovascular disease, non valvular	No	5 103 092	40 586	1.0 (ref)			
	Yes	27 508	206	1.14 (0.99–1.31)	0.06	1.16 (1.02–1.34)	0.03
Death due to non-rheumatic valvular disease	No	5 103 092	8057	1.0 (ref)			
	Yes	27 508	31	0.88 (0.62–1.26)	0.49	0.90 (0.64–1.29)	0.58
Death due to hypertension	No	5 103 092	8027	1.0 (ref)			
	Yes	27 508	43	1.23 (0.91–1.66)	0.18	1.30 (0.97–1.76)	0.08

*Adjusted for sex, birth year and number of first degree relatives. Analyses include individuals with diabetes mellitus as a contributing cause of death

**Table 4:** Risk of death due to categories of renal disease according to whether or not a first degree relative has end stage renal disease.

	Relative with ESRD	N total cohort	N deceased individuals	Hazard ratio	p-value	Adjusted hazard ratio*	p-value
Death due to all cause non-hereditary diseases of the kidneys and ureters	No	5 103 092	7094	1.0 (ref)			
	Yes	27 508	69	2.18 (1.72–2.77)	<0.001	2.29 (1.81–2.91)	<0.001
Death due to kidney failure of uncertain etiology	No	5 103 092	3971	1.0 (ref)			
	Yes	27 508	31	1.79 (1.26–2.55)	0.001	1.80 (1.26–2.56)	0.001
Death due to glomerular disease	No	5 103 092	1170	1.0 (ref)			
	Yes	27 508	27	4.79 (3.27–7.01)	<0.001	5.69 (3.88–8.34)	<0.001
Death due to interstitial renal disease	No	5 103 092	1484	1.0 (ref)			
	Yes	27 508	9	1.36 (0.71–2.62)	0.36	1.50 (0.78–2.89)	0.23
Death due to congenital or inherited renal disease	No	5 103 092	265	1.0 (ref)			
	Yes	27 508	54	41.2 (30.7–55.3)	<0.001	51.1 (37.9–68.9)	<0.001
Death due to “other” renal disease	No	5 103 092	469	1.0 (ref)			
	Yes	27 508	2	0.97 (0.24–3.87)	0.96	1.04 (0.26–4.17)	0.96

* Adjusted for sex, birth year and number of relatives. Analyses include individuals with diabetes mellitus as a contributing cause of death.

Data were also analyzed according to cause of ESRD in first degree relatives. aHR for death in individuals with a first degree relative with hypertensive nephropathy was 1.24 (1.14–1.34), while aHRs for death in individuals with first degree relatives with diabetic nephropathy or interstitial disease were 1.21 (1.13–1.29) and 1.12 (1.02–1.23) respectively(S2 Table). Interestingly, aHR for death in individuals whose first degree relatives had known hereditary renal disease was slightly smaller, 1.17 (1.08–1.28) while ESRD due to glomerular disease was not associated with an increase in aHR.

**Fig 3:**
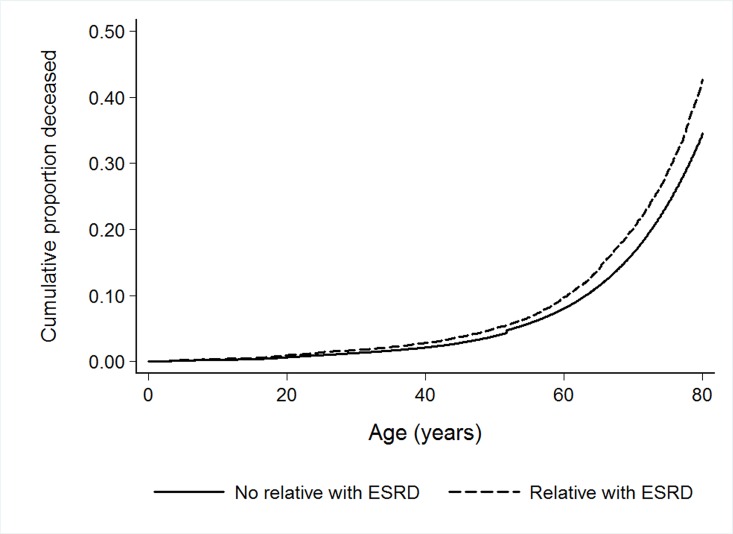
Adjusted Kaplan Meier failure curve for all cause death in individuals with and without a first degree relative with ESRD.

**Fig 4:**
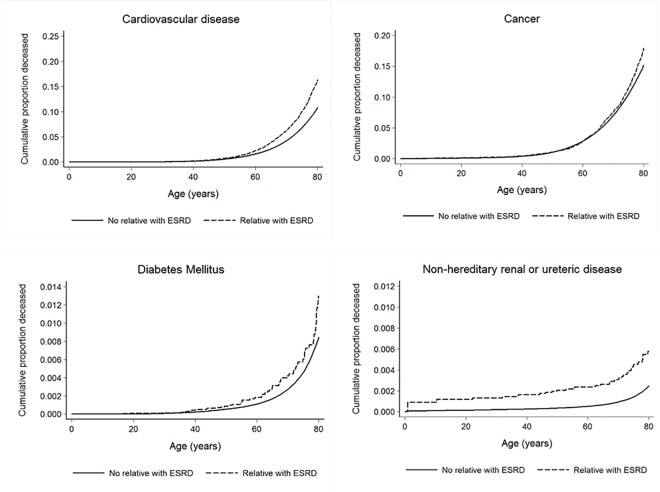
Adjusted Kaplan Meier failure curves for different categories of death according to whether or not a first degree relative had ESRD.

Absolute increases in mortality rates and adjusted hazard ratios between those with and without a relative with ESRD were further explored in cohorts defined by birth year (Table 5). These analyses showed that the absolute increases in mortality rates were highest in cohorts born 1900–1939, which was to a large degree explained by an increase in cardiovascular mortality, but a significant increase in mortality due to renal diseases were also seen. Interestingly, the cohort born 1940–1959 had a lower mortality rate if a relative had ESRD, this was mostly explained by a lower mortality rate due to cancer. In gender specific analyses, adjusted hazard ratios were higher for women born 1960–2009 and 1900–1919, aHR 1.38 (1.12–1.69) and 1.15 (1.07–1.22) respectively, compared to 1.12 (1.09–1.45) and 1.05 (0.98–1.11) for men born during the same time periods. Interestingly, the highest adjusted hazard ratio for cardiovascular death was seen in women born between 1940 and 1959, aHR 1.99 (1.46–2.67), identical analysis for men showed aHR of 1.25 (1.03–1.52) (S3 Table). Cumulative risk of death by age is also shown in Figs 3 and 4. Fig 3 shows that compared to individuals without a first degree relative with ESRD, individuals with a relative with ESRD were more likely to die at a younger age. From Fig 4 it is evident that the risk was higher if death was due to diseases of the kidneys or ureters, cardiovascular disease or diabetes mellitus.

**Table 5:** Relative and absolute risk of death for all cause death, cardiovascular death and renal death according to whether or not a first degree relative had ESRD and for subgroups of birth-year.

	Individuals without a first degree relative with ESRD			Individuals with a first degree relative with ESRD			Absolute mortality increase per 100 000 person-years (95% CI)	Adjusted Hazard ratio* (95% CI)
	N at risk	N death	Deaths per 100 000 person-years (95% CI)	N at risk	N death	Deaths per 100 000 person-years (95% CI)		
**All cause death**								
1960–2009	2 777 810	32 482	48.1 (47.6–48.7)	12 870	281	64.9 (57.5–72.7)	16.7 (9.1–24.3)	1.29 (1.15–1.46)
1940–1959	1 127 779	84 665	192 (191–193)	9 729	629	165 (152–178)	-27.1 (-40.0 - -14.1)	1.16 (1.08–1.26)
1920–1939	766 246	315 981	1179 (1175–1183)	2 845	1 297	1327 (1256.-1401)	148 (76–220)	1.17 (1.11–1.12)
1900–1919	431 257	390 789	3721 (3710–3733)	2 064	1 898	3939 (3764–4118)	218 (44–392)	1.09 (1.04–1.14)
**Cardiovascular death**								
1960–2009	2 777 810	1 864	2.76 (2.64–2.89)	12 870	21	4.85 (3.00–7.14)	2.1 (0.01–4.2)	1.33 (0.86–2.04)
1940–1959	1 127 779	16 547	37.5 (37.0–38.1)	9 729	143	37.5 (31.6–43.9)	-0.03 (-6.2–6.1)	1.41 (1.19–1.66)
1920–1939	766 246	121 374	453 (450–455)	2 845	515	527 (483–538)	74.2 (28.7–120)	1.21 (1.11–1.32)
1900–1919	431 257	198 525	1890 (1882–1899)	2 064	972	2017 (1893–2146)	127 (1.2–253)	1.10 (1.03–1.17)
**Renal death**								
1960–2009	2 777 810	68	0.10 (0.08–0.13)	12 870	3	0.69 (0.13–1.70)	0.59 (-0.19–1.38)	7.07 (2.21–22.6)
1940–1959	1 127 779	287	0.65 (0.58–0.73)	9 729	10	2.62 (1.25–4.50)	1.97 (0.34–3.60)	6.61 (3.50–12.5)
1920–1939	766 246	2 544	9.49 (9.13–9.86)	2 845	24	24.6 (15.7–35.4)	15.0 (5.2–24.9)	2.68 (1.79–4.00)
1900–1919	431 257	4 196	40.0 (38.8–41.2)	2 064	32	66.4 (45.4–91.4)	26.5 (3.4–49.5)	1.69 (1.19–2.40)
**Cancer**								
1960–2009	2 777 810	4 692	6.95 (6.76–7.15)	12 870	42	9.70 (6.99–12.9)	2.7 (-0.2–5.7)	1.19 (0.88–1.61)
1940–1959	1 127 779	31 483	71.4 (70.6–72.2)	9 729	187	49.0 (42.3–56.3)	-22.3 (-29.4 - -15.3)	0.95 (0.82–1.10)
1920–1939	766 246	105 366	393 (391–396)	2 845	380	389 (351–429)	-4.2 (-43.3–34.9)	1.02 0.92–1.12)
1900–1919	431 257	81 581	777 (772–782)	2 064	342	710 (637–787)	-67 (-142-8.1)	0.95 (0.85–1.06)

* Adjusted for sex and number of relatives. Analyses include individuals with diabetes mellitus as a contributing cause of death

## Discussion

In this population of predominantly European decent, the hazard ratio for all cause death was 13% higher in individuals who had a first degree relative with ESRD as compared to individuals who did not have a first degree relative with ESRD. The most important explanation was a higher risk of death due to cardiovascular disease, and cerebrovascular disease was associated with the highest hazard ratio. Hazard ratios for death due to kidney or urinary tract disease and diabetes mellitus were also significantly increased, about two-fold. Survival curves indicated that individuals with a first degree relative with ESRD were more likely to die at a younger age compared to individuals without a relative with ESRD, but the absolute increase in mortality rates were highest in the oldest cohorts born 1900–1939, where an increase in mortality of 295 per 100,000 person-years was seen.

Cardiovascular disease was the most common cause of death, the aHR of 1.15 (95% CI 1.10–1.21) for cardiovascular death in individuals with a first degree relative with ESRD was similar to the reported relative risk of 1.10 in a case control study from Utah[6]. Familial clustering of cardiovascular disease[10, 11] and increased risk of cardiovascular disease with declining GFR is well known[3, 4], diabetes mellitus is also a major risk factor for ESRD[12, 13] and known to aggregate in families[14, 15]. Analyses of different birth cohorts showed that the hazard ratio was highest with 1.41 for the cohort born 1940–1959, the excess risk seemed to be more pronounced in women. The incidence of smoking in Norwegian women increased after World War II, with gender convergence in tobacco use in the mid1970s[16], which may have impacted the risk associated with a family history of ESRD in women. The cohort born 1920–1939 had both a highly significant aHR and a highly significant increase in mortality rates, their mean age at death was 69.5 ±10.6 years. The fairly young age at death in this cohort, with the strongest increase in cardiovascular mortality, points towards premature cardiovascular death in individuals with a relative with ESRD. Cerebrovascular death was associated with the highest hazard ratio in the cardiovascular category in the Norwegian population, whereas cerebrovascular death was less common and not statistically significant in the Utah population[6]. Acute myocardial infarction and heart failure were the two main causes of cardiovascular death in the population from Utah. Differences in coding practices and study methodologies could probably explain some of these differences. In the current study hypertensive or diabetic ESRD in a first degree relative were associated with a 24% and 21% increase in risk of all cause death respectively, as compared to 13% when risk was analyzed irrespective of cause of ESRD in first degree relatives. Some of the increased risk may be attributable to shared environmental risk factors, or shared risk behavior such as smoking or diet. Having a relative with ESRD significantly increased the HR for death due to cerebrovascular/cardiovascular disease and diabetes mellitus, conversely hypertensive or diabetic ESRD in a first degree relative further increased the HR for all cause death. In our opinion these consistent findings add to previous reports, and strongly argue that shared genetic risk factors contribute to excess risk of death. The findings may also account for some of the excess risk of all cause death and cardiovascular death reported in Norwegian living kidney donors, most of whom were first degree relatives of the recipient[7].

Adjusted hazard ratio for death from non-hereditary diseases of the kidneys and/or ureters was 2.29 (1.81–2.91), of which aHR of death due to renal failure was 1.80 (1.26–2.56). This is significantly lower than findings described in a registry based study from Utah[1] where first degree relatives of ESRD patients of predominantly European decent had relative risks of dying with ESRD or non-ESRD CKD of 10.1 and 3.89, respectively. However, risk estimates of dying with ESRD or CKD were given, and ESRD or CKD may not have been the primary cause of death. This is in contrast to our study, in which only primary causes of death were included in the main analyses. Information on contributing causes of death was available for some, but not all individuals. Data on chronic kidney disease as a contributing cause of death were incomplete, and could in our opinion not be analyzed in this study. In our study, adjusted hazard ratio for death due to primary glomerular diseases was 5.69 (3.88–8.34) in individuals with a relative with ESRD. Disease specific causes of ESRD were not always verified by a kidney biopsy, and some cases were likely mislabeled as non-hereditary. Current knowledge of hereditary renal disease would have been unavailable for some of the follow-up period, and some cases, such as primary focal and segmental glomerulosclerosis, may actually have been hereditary[17, 18].

Somewhat unexpected, in individuals born 1940–1959, absolute cancer mortality was lower in individuals with a relative with ESRD as compared to individuals without a relative with ESRD. The reason for this observation is uncertain. It did not reach statistical significance in Cox regression analyses, and may be coincidental or due to coding practices In general, studies have shown increased risk of cancer in patients with kidney disease[19, 20] and several well-known risk factors of kidney disease, such as hypertension, inflammation, obesity, smoking and diet, have been shown to also be risk factors for cancer[21]. One might therefore expect an increased risk of cancer in relatives of patients with ESRD, but to our knowledge no studies have previously investigated this.

The strengths of this study include the nationwide cohort design and that complete national registry data were used to ascertain risk of death. The main limitations are that data are not complete for the whole follow-up period. Family data are complete for those born after 1952, and ESRD was not registered until 1980. Incomplete family data before 1953 would lead to an underestimation of the probability of having a first degree relative with ESRD, however it is unlikely to significantly have affected the estimates of excess risk. Outcomes were not registered until 1969, which was accounted for by the counting process modifications of the Cox regression statistics. This assumes that the effect of the predictor variable was mostly unchanged across time, i.e. excess risk attributed to having a relative with ESRD would be similar for a 40-year-old individual in 1980 and 2005. In our opinion this assumption is fair, and unlikely to have affected the results significantly. Adjusted hazard ratios are reported in order to eliminate confounding variables. Incidence of ESRD increases with age, and middle aged individuals would be more likely to have older first degree relatives with ESRD compared to younger individuals. Adjusted hazard ratios correct for older individuals being more likely to have a first degree relative with ESRD at the time of death. Absolute increases in mortality rates associated with having a first degree relative with ESRD were reported for different birth cohorts and causes of death. The decision to illustrate the cause-specific death rates for birth cohorts and not with traditional Kaplan Meier curves for the entire cohort was due to the fact that the different birth cohorts had different number of recorded relatives and different age-periods of follow-up. The findings for all cause death were in the same range as the overall analyses, except higher hazard ratios for the youngest cohort, confirming that the counting processes of Cox-regression chosen in the main analyses were adequate. Different editions of ICD codes were used across the follow-up period, it has not been possible to review and recode the diagnoses indicated on the death certificates. Information on diseases not directly linked to cause of death or disease specific risk factors were not available, and analyses could therefore not be adjusted for i.e smoking, hypercholesterolemia or background hypertension. Of note, mortality was only 95% at age 95 in the present study, and did not increase further, indicating a loss to follow-up likely due to 5% emigration, which is similar to the expected number from Statistics Norway. Emigration rate is unlikely to have affected the main results.

In conclusion, this study has quantified the excess risk of death associated with ESRD in a first degree relative in the Norwegian population. As expected cardiovascular death and chronic kidney disease related deaths were the main contributors to excess risk of death. Individuals who had a first degree relative with ESRD died at a younger age compared to those without a relative with ESRD. Excess risk of death in first degree relatives of ESRD patients may indicate shared genetic risk factors, highlighting the importance of focus on modifiable risk factors such as obesity, smoking, glycemic control and hypertension. Shared genetic risk factors within families may also account for some of the increased risk of death previously reported in Norwegian living kidney donors, most of whom were first degree relatives of the recipient.

## Supporting Information

S1 Appendix Dataset information. (DOC)

S1 Table Frequency for causes of death according to the European shortlist[22]. (DOC)

S2 Table Risk of death according to different causes of ESRD in first degree relatives. (DOC)

S3 Table Relative risk of death for all cause death, cardiovascular death and renal death according to gender and whether or not a first degree relative had ESRD and for subgroups of birth-year. (DOC)

## References

[1] Goldfarb-Rumyantzev AS, Cheung AK, Habib AN, Wang BJ, Lin SJ, Baird BC, et al A population-based assessment of the familial component of chronic kidney disease mortality. American journal of nephrology. 2006;26(2):142–8. Epub 2006/03/25. 10.1159/000092280. . 16557020

[2] Skrunes R, Svarstad E, Reisaeter AV, Vikse BE. Familial clustering of ESRD in the Norwegian population. Clinical journal of the American Society of Nephrology: CJASN. 2014;9(10):1692–700. Epub 2014/08/06. 10.2215/cjn.01680214 ; PubMed Central PMCID: PMCPmc4186510. 25092600 PMC4186510

[3] Go AS, Chertow GM, Fan D, McCulloch CE, Hsu C-y. Chronic kidney disease and the risks of death, cardiovascular events, and hospitalization. New England Journal of Medicine. 2004;351(13):1296–305. 10.1056/NEJMoa041031 15385656

[4] Sarnak MJ, Levey AS, Schoolwerth AC, Coresh J, Culleton B, Hamm LL, et al Kidney disease as a risk factor for development of cardiovascular disease a statement from the American Heart Association Councils on kidney in cardiovascular disease, high blood pressure research, clinical cardiology, and epidemiology and prevention. Circulation. 2003;108(17):2154–69. 10.1161/01.CIR.0000095676.90936.80 14581387

[5] Tonelli M, Wiebe N, Culleton B, House A, Rabbat C, Fok M, et al Chronic kidney disease and mortality risk: a systematic review. Journal of the American Society of Nephrology. 2006;17(7):2034–47. 10.1681/ASN.2005101085 16738019

[6] Naiman N, Cheung AK, Goldfarb-Rumyantzev AS. Familiality of cardiovascular mortality in end-stage renal disease patients. American journal of nephrology. 2009;29(3):237–43. Epub 2008/09/19. 10.1159/000156721 . 18799871

[7] Mjoen G, Hallan S, Hartmann A, Foss A, Midtvedt K, Oyen O, et al Long-term risks for kidney donors. Kidney Int. 2014;86(1):162–7. Epub 2013/11/29. 10.1038/ki.2013.460 . 24284516

[8] van Dijk PCW, Jager KJ, de Charro F, Collart F, Cornet R, Dekker FW, et al Renal replacement therapy in Europe: the results of a collaborative effort by the ERA–EDTA registry and six national or regional registries. Nephrology Dialysis Transplantation. 2001;16(6):1120–9. 10.1093/ndt/16.6.1120 11390709

[9] Andersen PK, Gill RD. Cox's regression model for counting processes: a large sample study. The annals of statistics. 1982:1100–20.

[10] Lloyd-Jones DM, Nam B-H, D'Agostino RB Sr, Levy D, Murabito JM, Wang TJ, et al Parental cardiovascular disease as a risk factor for cardiovascular disease in middle-aged adults: a prospective study of parents and offspring. Jama. 2004;291(18):2204–11. 10.1001/jama.291.18.2204 15138242

[11] Sesso HD, Lee I-M, Gaziano JM, Rexrode KM, Glynn RJ, Buring JE. Maternal and paternal history of myocardial infarction and risk of cardiovascular disease in men and women. Circulation. 2001;104(4):393–8. 11468199 10.1161/hc2901.093115

[12] Finne P, Reunanen A, Stenman S, Groop P, Grönhagen-Riska C. INcidence of end-stage renal disease in patients with type 1 diabetes. JAMA. 2005;294(14):1782–7. 10.1001/jama.294.14.1782 16219881

[13] Brancati FL, Whelton PK, Randall BL, Neaton JD, Stamler J, Klag MJ. Risk of end-stage renal disease in diabetes mellitus: A prospective cohort study of men screened for mrfit. JAMA. 1997;278(23):2069–74. 10.1001/jama.1997.03550230045035 9403420

[14] Association AD. Diagnosis and classification of diabetes mellitus. Diabetes care. 2010;33(Supplement 1):S62–S9. 20042775 10.2337/dc10-S062 PMC2797383

[15] Florez JC, Hirschhorn J, Altshuler D. The inherited basis of diabetes mellitus: implications for the genetic analysis of complex traits. Annual review of genomics and human genetics. 2003;4(1):257–91. 10.1146/annurev.genom.4.070802.110436 14527304

[16] Kjønstadutvalget. Tobakksindustriens erstatningsansvar Oslo, Norway: Seksjon statens trykning; 2000 [cited 2016 05.09]. 2000:16:[Available from: https://www.regjeringen.no/contentassets/8a1a5d1945d04f8799d2b5e85a9069c1/no/pdfa/nou200020000016000dddpdfa.pdf.

[17] Brown EJ, Pollak MR, Barua M. Genetic testing for nephrotic syndrome and FSGS in the era of next-generation sequencing. Kidney Int. 2014;85(5):1030–8. 10.1038/ki.2014.48 24599252 PMC4118212

[18] Pollak MR. Inherited podocytopathies: FSGS and nephrotic syndrome from a genetic viewpoint. Journal of the American Society of Nephrology. 2002;13(12):3016–23. 12444222 10.1097/01.asn.0000039569.34360.5e

[19] Wong G, Hayen A, Chapman JR, Webster AC, Wang JJ, Mitchell P, et al Association of CKD and Cancer Risk in Older People. Journal of the American Society of Nephrology. 2009;20(6):1341–50. 10.1681/asn.2008090998 19406977 PMC2689896

[20] Stewart JH, Buccianti G, Agodoa L, Gellert R, McCredie MR, Lowenfels AB, et al Cancers of the kidney and urinary tract in patients on dialysis for end-stage renal disease: analysis of data from the United States, Europe, and Australia and New Zealand. Journal of the American Society of Nephrology. 2003;14(1):197–207. 12506152 10.1097/01.asn.0000039608.81046.81

[21] Koene RJ, Prizment AE, Blaes A, Konety SH. Shared Risk Factors in Cardiovascular Disease and Cancer. Circulation. 2016;133(11):1104–14. Epub 2016/03/16. 10.1161/circulationaha.115.020406 26976915 PMC4800750

[22] Eurostat. European Shortlist Causes of Death http://ec.europa.eu/eurostat/ramon/documents/cod_1998/cod_1998_pdf.zip: Eurostat; 1998 [cited 2015 12.07.2015].

