# Supplementary material for: End Stage Renal Disease Predicts Increased Risk of Death in First Degree Relatives in the Norwegian Population
Source: PLoS One. 2016 Nov 9;11(11):e0165026. doi: 10.1371/journal.pone.0165026 (PMC5102372; doi:10.1371/journal.pone.0165026)
Supplement: S1 Appendix — (DOC) [file pone.0165026.s001.doc]

Dear Editors,

Due to the nature and size of the dataset we are apprehensive about uploading it to a digital repository. As the dataset comprises information from three nationwide registries on the whole Norwegian population, special care has to be taken so that no single individual can be identified (Norwegian laws and regulations estimate that such a dataset require that at least 5 persons must share all data). While preparing this dataset we observed that such a dataset had little value due to the categorizations that were needed. In addition we are, even after anonymization, uncomfortable uploading it due to the small size of the Norwegian population and the comprehensiveness of the dataset. We are however more than happy to make the dataset available for inspection on site, at Haukeland University Hospital.

We sincerely apologize for any inconvenience this may cause, and would like to stress that the dataset is indeed available for inspection, though not as an uploaded file due to the concerns outlined above.
