# Supplementary material for: End Stage Renal Disease Predicts Increased Risk of Death in First Degree Relatives in the Norwegian Population
Source: PLoS One. 2016 Nov 9;11(11):e0165026. doi: 10.1371/journal.pone.0165026 (PMC5102372; doi:10.1371/journal.pone.0165026)
Supplement: S1 Table — (DOC) [file pone.0165026.s002.doc]

S1 Table. Frequency for causes of death according to the European shortlist[19]

| Category number | Category name | Included ICD-10 diagnoses* | N deaths | % deaths |
| --- | --- | --- | --- | --- |
| E01 | Infectious diseases | A00-B99 | 9 220 | 1.11 |
| E06 | Neoplasms | C00-D48 | 228 676 | 27.62 |
| E25 | Diseases of the blood forming organs | D50-D89 | 1 680 | 0.20 |
| E26 | Endocrine. nutritional and metabolic diseases | E00-E90 | 14 355 | 1.73 |
| E33 | Diseases of the circulatory system | I00-I99 | 339 961 | 41.06 |
| E28 | Mental and behavioral disorders | F00-F99 | 17 881 | 2.16 |
| E31 | Diseases of the nervous system and the sense organs | G00-H95 | 19 972 | 2.41 |
| E37 | Diseases of the respiratory system | J00-J99 | 62 292 | 7.52 |
| E42 | Diseases of the digestive system | K00-K93 | 23 307 | 2.81 |
| E45 | Diseases of the skin and subcutaneous tissue | L00-L99 | 755 | 0.09 |
| E46 | Diseases of the musculoskeletal system/connective tissue | M00-M99 | 4 585 | 0.55 |
| E48 | Diseases of the genitourinary system | N00-N99 | 10 889 | 1.32 |
| E50 | Complications of pregnancy. childbirth and puerperium | O00-99 | 126 | 0.02 |
| E51 | Certain conditions originating in the perinatal period | P00-P96 | 63 | 0.01 |
| E52 | Congenital malformations and chromosomal abnormalities | Q00-Q99 | 2 801 | 0.34 |
| E55 | Symptoms. signs. abnormal findings. ill-defined causes | R00-R99 | 29 504 | 3.56 |
| E58 | External causes of injury and poisoning | V01-Y89 | 61 955 | 7.48 |

*Only ICD-10 codes given as example of included diagnoses. Same diagnoses included from ICD 8 and 9. See European shortlist for details
