# Supplementary material for: End Stage Renal Disease Predicts Increased Risk of Death in First Degree Relatives in the Norwegian Population
Source: PLoS One. 2016 Nov 9;11(11):e0165026. doi: 10.1371/journal.pone.0165026 (PMC5102372; doi:10.1371/journal.pone.0165026)
Supplement: S2 Table — (DOC) [file pone.0165026.s003.doc]

S2 Table. Risk of death according to different causes of ESRD in first degree relatives

|  | Relative with ESRD | N total cohort | N deceased individuals | Hazard ratio | p value | Adjusted hazard ratio | p value |
| --- | --- | --- | --- | --- | --- | --- | --- |
| Relative with glomerular disease | No | 5 208 588 | 843 123 | 1.0 (ref) |  | 1.0 (ref) |  |
|  | Yes | 8 980 | 1 284 | 1.00 (0.94-1.05) | 0.94 | 1.06 (1.00-1.12) | 0.06 |
|  |  |  |  |  |  |  |  |
| Relative with interstitial disease | No | 5 214 699 | 843 982 | 1.0 (ref) |  | 1.0 (ref) |  |
|  | Yes | 2 869 | 425 | 1.04 (0.95-1.15) | 0.38 | 1.12 (1.02-1.23) | 0.02 |
|  |  |  |  |  |  |  |  |
| Relative with hereditary disease | No | 5 214 107 | 843 870 | 1.0 (ref) |  | 1.0 (ref) |  |
|  | Yes | 3 461 | 537 | 1.14 (1.05-1.24) | 0.002 | 1.17 (1.08-1.28) | <0.001 |
|  |  |  |  |  |  |  |  |
| Relative with diabetic nephropathy | No | 5 213 513 | 843 556 | 1.0 (ref) |  | 1.0 (ref) |  |
|  | Yes | 4 055 | 851 | 1.17 (1.10-1.25) | <0.001 | 1.21 (1.13-1.29) | <0.001 |
|  |  |  |  |  |  |  |  |
| Relative with hypertensive nephropathy | No | 5 212 844 | 843 804 | 1.0 (ref) |  | 1.0 (ref) |  |
|  | Yes | 4 724 | 603 | 1.15 (1.06-1.25) | 0.001 | 1.24 (1.14-1.34) | <0.001 |
|  |  |  |  |  |  |  |  |
| Relative with “other” renal disease | No | 5 214 007 | 843 897 | 1.0 (ref) |  | 1.0 (ref) |  |
|  | Yes | 3 561 | 510 | 1.03 (0.95-1.13) | 0.48 | 1.10 (1.00-1.20) | 0.04 |

Adjusted for sex. birth year. number of first degree relatives
