# Supplementary material for: End Stage Renal Disease Predicts Increased Risk of Death in First Degree Relatives in the Norwegian Population
Source: PLoS One. 2016 Nov 9;11(11):e0165026. doi: 10.1371/journal.pone.0165026 (PMC5102372; doi:10.1371/journal.pone.0165026)
Supplement: S3 Table — (DOC) [file pone.0165026.s004.doc]

S3 Table. Relative risk of death for all cause death, cardiovascular death and renal death according to gender and whether or not a first degree relative had ESRD and for subgroups of birth-year

|  | Males with a first degree relative with ESRD | | |  |  |  | Females with a first degree relative with ESRD | | |  |  |
| --- | --- | --- | --- | --- | --- | --- | --- | --- | --- | --- | --- |
| **All cause death** | N deceased | aHR (95% CI)* | P value | HR (95% CI) | p-value |  | N deceased | aHR (95% CI)* | P value | HR (95% CI) | P value |
| 1960-2009 | 188 | 1.26 (1.09-1.45) | 0.002 | 1.12 (0.97-1.29) | 0.117 |  | 93 | 1.38 (1.12-1.69) | 0.002 | 1.25 (1.02-1.53) | 0.035 |
| 1940-1959 | 421 | 1.17 (1.06-1.29) | 0.001 | 1.00 ( 0.91-1.11) | 0.923 |  | 208 | 1.15 (1.00-1.32) | 0.044 | 1.02 (0.89-1.17) | 0.761 |
| 1920-1939 | 756 | 1.19 (1.11-1.28) | <0.001 | 1.14 (1.06-1.23) | <0.001 |  | 541 | 1.14 (1.04-1.24) | 0.003 | 1.10 (1.01-1.19) | 0.032 |
| 1900-1919 | 980 | 1.05 (0.98-1.11) | 0.155 | 1.03 (0.96-1.09) | 0.400 |  | 916 | 1.15 (1.07-1.22) | <0.001 | 1.12 (1.05-1.19) | 0.001 |
| **Cardiovascular death** |  |  |  |  |  |  |  |  |  |  |  |
| 1960-2009 | 12 | 1.09 (0.62-1.93) | 0.761 | 1.02 (0.59-1.80) | 0.940 |  | 9 | 1.86 (0.96-3.60) | 0.064 | 1.74 (0.90-3.37) | 0.098 |
| 1940-1959 | 100 | 1.25 (1.03-1.52) | 0.025 | 1.09 (0.89-1.32) | 0.404 |  | 43 | 1.99 (1.46-2.67) | <0.001 | 1.73 (1.28-2.34) | <0.001 |
| 1920-1939 | 326 | 1.23 (1.10-1.37) | <0.001 | 1.18 (1.06-1.32) | 0.003 |  | 189 | 1.17 (1.02-1.35) | 0.029 | 1.14 (0.99-1.31) | 0.075 |
| 1900-1919 | 513 | 1.07 (0.99-1.17) | 0.105 | 1.06 (0.97-1.15) | 0.224 |  | 458 | 1.13 (1.03-1.24) | 0.011 | 1.11 (1.01-1.21) | 0.029 |
| **Renal death** |  |  |  |  |  |  |  |  |  |  |  |
| 1960-2009 | 2 | 6.74 (1.63-27.9) | 0.009 | 5.85 (1.41-24.17) | 0.015 |  | 1 | 7.86 (1.05-58.95) | 0.045 | 6.84 (0.92-51.0) | 0.061 |
| 1940-1959 | 5 | 5.60 (2.28-13.72) | <0.001 | 4.16 (1.71-10.14) | 0.002 |  | 5 | 8.13 (3.29-20.06) | <0.001 | 5.62 (2.30-13.8) | <0.001 |
| 1920-1939 | 12 | 2.42 (1.37-4.27) | 0.002 | 2.33 (1.32-4.11) | 0.004 |  | 12 | 3.00 (1.70-5.31) | <0.001 | 2.85 (1.61-5.03) | <0.001 |
| 1900-1919 | 15 | 1.49 (0.90-2.48) | 0.122 | 1.49 (0.89-2.47) | 0.127 |  | 17 | 1.92 (1.19-3.09) | 0.008 | 1.91 (1.18-3.07) | 0.008 |
| **Cancer death** |  |  |  |  |  |  |  |  |  |  |  |
| 1960-2009 | 19 | 1.15 (0.73-1.81) | 0.583 | 1.06 (0.68-1.67) | 0.768 |  | 23 | 1.22 (0.81-1.84) | 0.337 | 1.16 (0.77-1.75) | 0.480 |
| 1940-1959 | 99 | 1.00 (0.82-1.21) | 0.971 | 0.92 (0.76-1.12) | 0.420 |  | 88 | 0.90 (0.73-1.11) | 0.336 | 0.83 (0.68-1.03) | 0.087 |
| 1920-1939 | 210 | 1.06 (0.92-1.21) | 0.413 | 1.03 (0.88-1.18) | 0.693 |  | 170 | 0.97 (0.84-1.13) | 0.719 | 0.94 (0.81-1.09) | 0.412 |
| 1900-1919 | 180 | 0.84 (0.73-0.97) | 0.020 | 0.82 (0.71-0.95) | 0.010 |  | 162 | 1.11 (0.95-1.29) | 0.195 | 1.06 (0.91-1.24) | 0.431 |

*Adjusted for number of first degree relatives
